# Mitochondrial DNA mutations are associated with impaired oxidative phosphorylation and enhanced imatinib response in chronic myeloid leukemia

**DOI:** 10.1002/hem3.70429

**Published:** 2026-09-07

**Authors:** Ilaria S. Pagani, Vaidehi Krishnan, John F. Ouyang, Chung H. Kok, BeiJun Chen, Kian Leong Lee, Cong M. Pham, Paul Wang, Elyse Page, Phuong Dang, Verity A. Saunders, Jane James, Kelly Lim, Stefano Mangiola, Yingnan Gao, Naranie Shanmuganathan, Agnes Yong, Susan Branford, Charles Chuah, David T. Yeung, Deborah L. White, Vignir G. Helgason, Daniel Thomas, S. Tiong Ong, Timothy P. Hughes, David M. Ross

**Affiliations:** ^1^ Precision Cancer Medicine Theme, Blood Cancer Program South Australian Health & Medical Research Institute (SAHMRI) Adelaide South Australia Australia; ^2^ School of Medicine, College of Health Adelaide University Adelaide South Australia Australia; ^3^ Australasian Leukaemia and Lymphoma Group Melbourne Victoria Australia; ^4^ Department of Medical Biology University of Melbourne Melbourne Victoria Australia; ^5^ Centre for Computational Biology, Program in Cardiovascular and Metabolic Disorders Duke‐NUS Medical School Singapore Singapore; ^6^ Centre for Cancer Biology University of South Australia Adelaide South Australia Australia; ^7^ Centre for Computational Biology, Duke‐NUS Medical School Singapore Singapore; ^8^ Bioinformatics and Computational Biology Division Walter and Eliza Hall Institute of Medical Research Melbourne Victoria Australia; ^9^ South Australian immunoGENomics Cancer Institute University of Adelaide Adelaide South Australia Australia; ^10^ Royal Perth Hospital and the University of Western Australia Medical School Perth Western Australia Australia; ^11^ Department of Haematology Singapore General Hospital and National Cancer Centre Singapore Singapore Singapore; ^12^ Wolfson Wohl Cancer Research Centre, School of Cancer Sciences University of Glasgow Glasgow United Kingdom; ^13^ Cancer and Stem Cell Biology Signature Research Programme Duke‐NUS Medical School Singapore Singapore; ^14^ Department of Haematology Singapore General Hospital Singapore Singapore; ^15^ Division of Medical Oncology Duke University Medical Center Durham North Carolina United States; ^16^ Department of Haematology and Genetic Pathology Flinders University and Medical Centre Adelaide South Australia Australia

## Abstract

Mitochondrial DNA (mtDNA) mutations are frequently observed in cancer, but their clinical and functional significance in chronic myeloid leukemia (CML) remains incompletely defined. Here, we show that a distinct mtDNA mutational landscape is associated with mitochondrial metabolic programs and response to imatinib therapy in CML. We performed comprehensive profiling of somatic mtDNA mutations in 120 patients with chronic‐phase CML. At diagnosis, 241 somatic mtDNA mutations were identified in 92 patients, including 29 homoplasmic mutations. In a clinically annotated cohort of 79 imatinib‐treated patients, a higher number of mtDNA mutations (≥3 mutations) and higher variant allele frequency were associated with superior molecular responses, and remained significant in multivariable analyses. mtDNA mutational patterns were associated with distinct metabolic phenotypes in CD34^+^ leukemic stem/progenitor cells. Suboptimal responders exhibited increased mitochondrial respiration, spare respiratory capacity, mitochondrial content, and enrichment of mitochondrial biogenesis and lipid metabolic programs, consistent with enhanced oxidative phosphorylation dependence. In contrast, favorable responders displayed higher mtDNA mutational burden together with reduced respiratory reserve and increased mitophagy‐related programs. Pharmacologic Complex I inhibition reduced clonogenic potential and enhanced imatinib sensitivity. Collectively, these findings identify mtDNA mutational states as a biomarker of metabolic fitness and therapeutic response in CML, while supporting further investigation of mitochondrial metabolism as a potential therapeutic vulnerability in CML.

## Graphical abstract

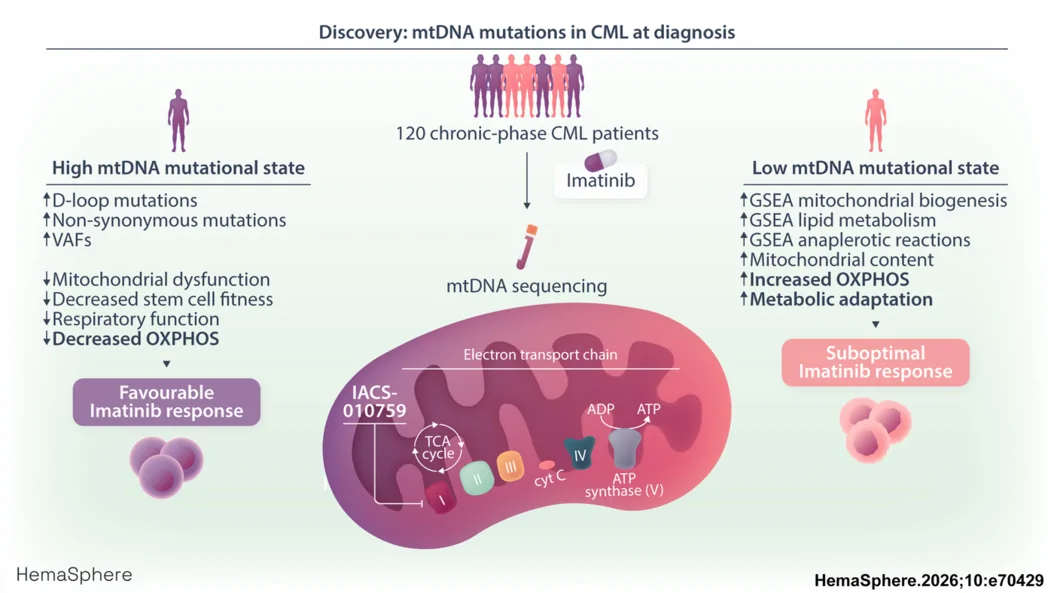

## INTRODUCTION

Chronic myeloid leukemia (CML) represents a paradigm of successful targeted therapy, driven by the BCR::ABL1 fusion protein tyrosine kinase.^1^, ^2^ Tyrosine kinase inhibitors (TKIs) have transformed CML from a fatal disease into a chronic condition, with 5‐year survival exceeding 80%.^3^ However, TKI resistance and disease progression to blast crisis remain major contributors to CML‐related mortality,^4^ and only about 25% of all CML patients can cease their TKI, achieving treatment‐free remission. The remaining patients require lifelong TKI therapy to prevent relapse, leading to organ toxicities, poor drug adherence, and reduced quality of life.^5^, ^6^

Resistance to TKIs is multifactorial,^7^ encompassing *BCR::ABL1* kinase domain mutations,^8^, ^9^ acquisition of additional genetic abnormalities,^10^, ^11^, ^12^ *BCR::ABL1* overexpression, activation of alternative signaling pathways,^13^ and protective interactions within the bone marrow microenvironment.^14^, ^15^ Increasingly, metabolic reprogramming has emerged as a key feature of CML persistence,^16^ with leukemic stem and progenitor cells (LSPCs) exhibiting enhanced oxidative phosphorylation (OXPHOS), which supports survival under TKI pressure and contributes to minimal residual disease.^17^, ^18^, ^19^, ^20^

**Figure 1:**
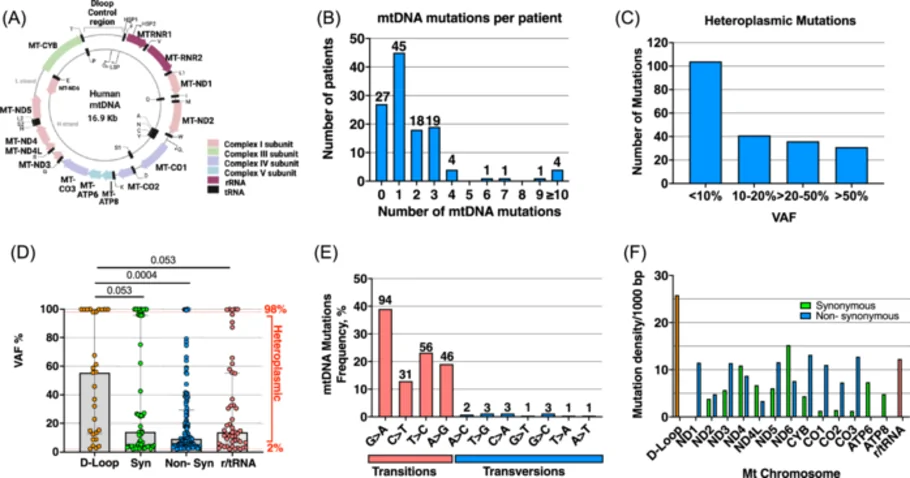
**Mitochondrial DNA (mtDNA) mutations in chronic myeloid leukemia (CML) patients at diagnosis. (A)** Schematic representation of the circular mtDNA genome. The figure was generated with *Biorender.com*. **(B)** Number of mtDNA somatic mutations per patient identified in 120 CML patients at diagnosis. **(C)** Variant allele frequency (VAF) distribution of somatic heteroplasmic mtDNA mutations. **(D)** VAF of mutations across different regions of the mitochondrial genome. Mutations with VAF greater than 98% are considered homoplasmic, and mutations with VAFs < 98% heteroplasmic. Mutations with VAF < 2% were empirically considered as sequencing error.^22^ (Kruskal–Wallis test; median with interquartile range). **(E)** Frequency of mutation types across the mtDNA genome. The majority of mutations were transitions (red), with G:C>A:T mutations comprising 39% and T:A>C:G mutations 23%. A smaller proportion of mutations were transversions (blue). **(F)** Mutation density per 1000 bp across the mitochondrial genome. The mutation rate per 1000 bp was calculated by dividing the number of mutations/gene by the gene length (gene‐length–normalized). The plot shows the mutation rates for various mtDNA regions, including the D‐loop (orange), structural RNAs (ribosomal RNA [rRNA] and transfer RNA [tRNA], red), and coding regions, highlighting the relative mutation frequencies across these areas. Mutations in the coding region were further subdivided in synonymous (green) and non‐synonymous (blue).

Mitochondria are central regulators of OXPHOS, redox homeostasis, and apoptotic signaling.^21^ The OXPHOS machinery, which is integral to the mitochondrial (mt) electron transport chain (ETC), is governed by dual genomic control: while the nuclear genome encodes the majority of OXPHOS proteins, the mitochondrial genome encodes 13 essential components of Complexes I and III–V (ATP synthase), alongside 22 transfer RNAs (tRNAs) and two ribosomal RNAs (rRNAs) (Figure 1A).^23^ MtDNA is a circular, double‐stranded molecule of approximately 16.6 kilobases (kb), which, unlike the nuclear genome, exists in multiple copies per mitochondrion, with each cell containing hundreds to thousands of mtDNA molecules.^24^ It can accumulate somatic mutations, leading to heteroplasmy, the co‐existence of wild‐type and mutant mitochondrial genomes within the same cell, whilst homoplasmy is the presence of identical mtDNA molecules within a cell.^24^

Although not traditionally regarded as oncogenic drivers, mtDNA mutations are increasingly recognized for their capacity to influence cancer progression and treatment response.^25^, ^26^, ^27^ Heteroplasmic mutations affecting protein‐coding regions of the ETC and the regulatory D‐loop region have been reported across diverse malignancies,^25^, ^28^, ^29^ where they contribute to altered OXPHOS, redox imbalance, and metabolic rewiring.^30^, ^31^ These alterations can enhance cellular adaptability to stress and influence therapeutic response.^30^, ^32^, ^33^, ^34^, ^35^, ^36^ However, the role of mtDNA mutations in CML remains largely unexplored.

In this study, we performed an integrated analysis of somatic mtDNA mutations at diagnosis of chronic‐phase (CP) CML, combining bulk sequencing of peripheral blood leukocytes, single‐cell transcriptomics, and functional profiling of LSPCs. We show that somatic mtDNA mutations are highly prevalent at diagnosis and that mtDNA mutational burden, heteroplasmy, and genomic distribution differ between patients with favorable versus suboptimal responses to imatinib. These differences were accompanied by distinct transcriptional and metabolic features, including altered mitochondrial respiration and biogenesis and lipid metabolic signatures in LSPCs. Together, our findings suggest that mtDNA mutations are associated with biologically distinct metabolic states linked to treatment response in CML and may provide a framework for patient stratification and metabolism‐directed therapeutic approaches.

## METHODS

### Patient characteristics

We retrospectively analyzed 120 CP‐CML patients treated with imatinib^37^, ^38^ (*n* = 79) or nilotinib^39^, ^40^ (*n* = 41).^39^, ^40^ Baseline characteristics are summarized in Supporting Information S2: Table 1. CP‐CML patients were broadly classified according to the European Leukemia Net recommendations^41^ into three categories: favorable responders (Group A), defined as achieving major molecular response (MMR; *BCR::ABL1* ≤ 0.1% international scale, IS)^42^ at 12 months; suboptimal responders (Group B), defined as not achieving the MMR milestone at 12 months; and treatment failure/progression (Group C) defined by progression to blast crisis. Among imatinib‐treated patients, 43 were classified as favorable, 33 as suboptimal, and 3 as progressive disease, of whom two progressed to myeloid blast crisis (#P0074 and # P0137) and one to central nervous system lymphoid blast crisis (#P0175). In the nilotinib cohort, 30 patients were classified as favorable and 11 as suboptimal.

### Primary samples

Total leukocytes or mononuclear cells were isolated from the peripheral blood of CML patients at diagnosis and after 12 months of TKI therapy,^22^ with informed consent obtained in accordance with the Declaration of Helsinki and approval of Central Adelaide Local Health Network (HREC/14/RAH/316). Normal samples were obtained from cryopreserved apheresis products of healthy G‐CSF‐mobilized donors.

CD34^+^ cells were isolated from peripheral blood CML and G‐CSF‐mobilized mononuclear cells using the CD34 MicroBead Kit (Miltenyi Biotec, Germany) and manual MACS Cell Separation (Miltenyi Biotec, Germany). Following MACS separation, CD34^+^ cells were cryopreserved for further analysis. Patient characteristics and samples included in functional experiments (Adelaide cohort) are summarized in Supporting Information S2: Table 2.

### Cell culture

CD34^+^ cells (2 × 10^5^ per well) were cultured in 24‐well plates at 37°C and 5% CO_2_ in serum‐free media (SFM) consisting of Iscove's modified Dulbecco medium (Sigma‐Aldrich, Missouri, USA) supplemented with BIT 9500 Serum Substitute (bovine serum albumin, BSA/insulin/transferrin; STEMCELL Technologies, Vancouver, Canada), 2 mM l‐glutamine (Sigma‐Aldrich, St. Louis, Missouri, USA), 1 mM streptomycin/penicillin, 0.1 mM 2‐mercaptoethanol, and 0.02 mg/mL human low‐density lipoprotein (STEMCELL Technologies, Vancouver, Canada). The SFM was then filtered and supplemented with a physiological growth factor cocktail (STEMCELL Technologies, Vancouver, Canada) consisting of 0.05 ng/mL human recombinant LIF, 1 ng/mL G‐CSF, and 0.2 ng/mL SCF/GM‐CSF/MIP‐α/IL‐6.

### Drug treatment

CD34^+^ cells were incubated with 2 µM Imatinib (kindly donated by Novartis, Basel, Switzerland) and 100 nM IACS‐010759 (Selleck Chemicals LLC, Texas, USA),^43^ either individually or in combination. IACS‐010759 was reconstituted in DMSO prior to use.

### Oxygen consumption rate measurement

Oxygen consumption rate (OCR) was measured using the Seahorse XF96 Analyzer (Agilent Technologies, California, USA) with the Seahorse XF Cell Mito Stress Test Kit (Agilent, California, USA). Primary cells were cultured overnight in SFM with physiological growth factors and then resuspended in warm Seahorse XF RPMI medium (pH 7.4), supplemented with 10 mM glucose, 1 mM pyruvate, and 2 mM glutamine (all from Agilent Technologies, California, USA). Eight replicates were performed per sample.

Cells (1.2 × 10^5^ per well) were seeded on Cell‐Tak‐coated plates and centrifuged at 800 rpm for 3 min, followed by a 1‐h incubation at 37°C in a non‐CO_2_ incubator. OCR was recorded at baseline and after sequential injections of oligomycin (1.5 μM), FCCP (2 μM), and rotenone plus antimycin A (0.5 μM each). Measurements were normalized to cell number. Additional details are provided in Supporting Information S1: Supplementary Methods.

### Mitochondrial content and membrane potential

Mitochondrial content and membrane potential were assessed using MitoTracker Green FM and MitoTracker Red CMXRos (Invitrogen Carlsbad, California) fluorescent probes, following the manufacturers' instructions.Additional details are provided in Supporting Information S1: Supplementary Methods.

### Colony forming cell assays

Primary cells were plated in SFM supplemented with PGF cocktail in the presence of the indicated drugs. After 3 days, cells from each condition were transferred in methylcellulose‐based medium (MethoCult H4034 Optimum, STEMCELL Technologies) in duplicate, and colonies were manually counted after 12–14 days.

### Next‐generation sequencing of the entire mitochondrial genome

MtDNA mutations (Supporting Information S2: Table 3) were identified as previously described.^22^ Briefly, genomic DNA was extracted using a phenol/chloroform method. The full 16.9 kb mitochondrial genome was amplified by long‐range polymerase chain reaction (PCR) using overlapping primer sets, and pooled amplicons were subjected to library preparation using the NexteraXT kit (Illumina, San Diego, CA) and sequenced on an Illumina MiSeq platform. Detailed PCR conditions, primer sequences, and purification steps are provided in the Supporting Information S1: Supplementary Methods.

### Calling of mtDNA variants and somatic mutations

Paired‐end mitochondrial reads were aligned to the Revised Cambridge Reference Sequence (rCRS, NC_012920), which includes a 3107 N deletion to preserve historical position numbering. Alignment was performed using bwa‐mem2 v2.2.1, and duplicate reads were marked using PicardTools v3.2.0. Variant calling in leukocytes at diagnosis was conducted with LoFreq v2.1.5^44^ in somatic mode. For patients with *BCR::ABL1* < 1% IS at 12 months (*n* = 116), the 12‐month peripheral blood sample was used as the germline control, as previously described.^22^ For the remaining four patients, matched non‐hematopoietic tissue (hair follicles or mesenchymal stem cells) was used as the germline controls (*n* = 4).

Functional annotation of variants was performed using the Ensembl Variant Effect Predictor (VEP) web interface, including predictions from SIFT^45^ and PolyPhen‐2.^46^ We previously empirically identified a cutoff of 2% to discriminate between sequencing errors (false positive) and variants at low variant allele frequency (VAF).^22^ All variants with VAF < 2% were disregarded; variants with VAF 2%–98% were considered as heteroplasmic, variants with VAF ≥ 98% were considered as homoplasmic, and at least five supporting reads per pair‐end were required. Several errors were removed from the final list of variants. They included the false positives C3106A, AC3105A, and N3107C generated by misalignment due to the 3107 N in the rCRS; the homopolymer tract in the region 302–315 generating the recurrent T310C and T310TC misalignments; and the T333C and T344C recurrent artefacts. Single‐nucleotide polymorphisms (SNPs) were also excluded.

### MtDNA copy number quantification

Relative mtDNA copy number was quantified by real‐time quantitative PCR (qPCR) using two mitochondrial gene targets, *mitochondrially encoded NADH dehydrogenase 1 (MT‐ND1*; Hs02596873‐s1, FAM‐MGB) and *mitochondrially encoded cytochrome b* (*MT‐CYB;* Hs02596867‐s1, FAM‐MGB), normalized to the nuclear reference gene *glucuronidase beta* (*GUSB*).^47^ Reactions were performed in triplicate using TaqMan assays on a QuantStudio™ 7 Flex system (Life Technologies, California, USA). The relative copy number per diploid genome was calculated using the ΔCt method, averaging results from both mitochondrial targets. Detailed primers, probes, reaction conditions, and calculation formulae are provided in the Supporting Information S1: Supplementary Methods.

### Gene‐set enrichment analysis and mtDNA variant analysis from single‐cell RNA sequencing data

We analyzed the 10 × 5′ single‐cell RNA sequencing (scRNA‐seq) dataset generated by Krishnan et al.^13^ comprising diagnostic bone marrow CD34^+^ cells from 24 CP‐CML patients and 8 healthy donors. CP‐CML patients were stratified according to the response categories defined above as Group A (favorable molecular response; *n* = 9), Group B (suboptimal molecular response; *n* = 9), and Group C (blast crisis/progression; *n* = 6; Supporting Information S2: Table 4).

Gene‐set enrichment analysis (GSEA) release 4.3.2 was used to perform 1000 gene‐set permutations where weighted enrichment statistics were calculated, and the signal‐to‐noise ratio was used as the metric for ranking genes (Supporting Information S2: Table 5). Enrichment scores were normalized using meandiv in comparisons of CD34^+^ LSPCs from different patient groups. The significance of normalized enrichment scores was determined based on the false discovery rate *q*‐value (≤0.25, significant; ≤0.05, highly significant). Gene sets of interest were curated from MSigDB v7.5.1. For gene sets with positive or negative NES scores, respectively, leading edge genes were determined before and after the peak where the running sum reaches the maximum deviation from zero.

Mitochondrial reads were extracted from the scRNA‐seq dataset to enable downstream single‐cell mtDNA variant analysis by using the Mitochondrial Genome Analysis Toolkit (mgatk).^48^ mgatk was applied to aligned BAM files generated from 10x Genomics data to detect mtDNA variants and quantify allele frequencies across individual cells (pipeline details are provided in the Supporting Information S1: Supplementary Methods). The resulting objects were imported into the Signac package (v1.14)^49^ for downstream analysis. Variant coverage was determined using the *IdentifyVariants* function, retaining variants detected with high confidence in at least two cells (n_cells_conf_detected ≥2). Single‐cell allele frequencies were calculated using the *AlleleFreq* function, and cells were considered to harbor a variant if the allele frequency exceeded 0.1. The median sequencing coverage per cell was 17.7 reads, resulting in a total of 957,796 mtDNA‐specific reads mapped across all cells. We identified and annotated mtDNA variants across 54,041 bone marrow cells, facilitating comprehensive analysis of variant prevalence and heterogeneity among patient groups. To assess lineage‐specific variant enrichment, we compared the number of mtDNA variants detected in hematopoietic stem and progenitor cells (HSPCs) with those observed in T and NK cells. Because sequencing depth can influence variant detection, the number of variants in HSPCs was normalized to that in T&NK cells by calculating an HSPC‐to‐T&NK variant ratio for each patient. Variants with VAF > 90% in T&NK cells were considered likely germline and excluded from the analysis. The HSPC‐to‐T&NK variant ratio was calculated for each patient and compared across clinical response groups. Using this normalization, we identified a total of 429 mtDNA variants across 41,290 LSPCs, with a median of 96 variants per patient (Supporting Information S2: Table 6). Variants were further classified as synonymous, non‐synonymous, or non‐coding, and category‐specific ratios were calculated. For visualization of cell populations in which mtDNA variants were assessed, uniform manifold approximation and projection (UMAP) embeddings were generated from the top 50 principal components of highly variable genes using Seurat (v5.1)^50^ workflow.

### Validation of mtDNA variants between total leukocytes and matched CD34^+^ LSPCs, and between bulk DNA sequencing and scRNA‐seq

We validated mtDNA mutations identified in total white cells by confirming their presence in matched bulk CD34^+^ LSPCs. We also compared variant calls from 5′ scRNA‐seq data to matched targeted bulk mtDNA sequencing. Detailed methodology, variant calling performance metrics, and results are provided in the Supporting Information S1: Supplementary Methods and Supporting Information S2: Tables 7 and 8.

### Statistical analysis

Comparisons between groups were performed using Fisher's exact test for categorical variables, and the Mann–Whitney *U* test or Kruskal–Wallis test for non‐normally distributed continuous variables. For normally distributed data, ordinary one‐way analysis of variance (ANOVA) was applied. Where applicable, the Bonferroni correction was used to adjust for multiple comparisons.

Kaplan–Meier estimates and log‐rank tests were used to calculate overall survival (OS) and failure‐free survival (FFS). FFS was defined according to ELN criteria,^41^ including failure to achieve time‐dependent molecular milestones, acquisition of *BCR::ABL1* kinase domain mutations, progression to accelerated or blast phase, or death from any cause. Progression to accelerated phase or blast crisis was additionally analyzed as a separate endpoint, and its cumulative incidence was assessed using Fine–Gray modeling. Similarly, the cumulative incidence of achieving MMR, MR4 (*BCR::ABL1* ≤ 0.01% IS), and MR4.5 (*BCR::ABL1* ≤ 0.0032% IS) was evaluated, stratified by number of mtDNA mutations, and mutations in D‐loop and *mitochondrially encoded cytochrome c oxidase I* (*MT‐CO1*).

Fine–Gray competing risks regression was performed for univariate and multivariate analyses to evaluate factors associated with achieving MMR and MR4.0 (*BCR::ABL1* ≤ 0.01% IS). Baseline variables analyzed included age at screening (continuous and dichotomized ≥50 vs. <50 years), sex, *BCR::ABL1* transcript type, Sokal score (categorical, with low risk as the reference category), spleen size (continuous), halving time (HT) (continuous),^51^ as well as number of mtDNA mutations (dichotomized), and the presence of D‐loop or *MT‐CO1* mutations (yes/no). Significant variables in univariate Fine–Gray analyses were assessed for independence before inclusion in multivariate models. Pairwise correlations between the focal variable (i.e., number of mtDNA mutations) and other potential confounders were evaluated using Spearman rank‐correlation tests for numerical variables and chi‐square tests for categorical variables. Variables without strong correlations were included in the multivariate models, and variance inflation factors were calculated to further assess multicollinearity. The overall significance of each multivariate model was assessed using a global Wald test.

All statistical analyses were conducted using GraphPad Prism v9.0 (GraphPad Software, USA) and R v4.2.0 (R Foundation for Statistical Computing, Austria). All P‐values were two‐sided, and values less than 0.05 were considered statistically significant.

## RESULTS

### mtDNA mutations are highly prevalent in total leukocytes of CML patients at diagnosis

To assess the landscape of mitochondrial genome alterations in CML, we analyzed somatic mtDNA point mutations in peripheral blood leukocytes from 120 CP‐CML patients at diagnosis.^22^ The complete list of mtDNA somatic point mutations is provided in Supporting Information S2: Table 3. We identified 241 somatic mutations in 92 patients (77%) (Supporting Information S2: Table 3), with a median of 1 mutation per patient (range 0–21; Figure 1B). Four patients harbored ≥10 mutations. Most mutations were present at low VAF (<10%, median 4.2%), suggesting heteroplasmy or sub‐clonal distribution,^24^, ^52^ whereas 29 mutations were homoplasmic (VAF ≥ 98%; Figure 1C).

The distribution of somatic point mutations across the mitochondrial genome is shown in Figure 1D. The majority were non‐synonymous coding variants (44%), with additional contributions from synonymous (24%), D‐loop (12%), and rRNA/tRNA (21%) mutations. Non‐coding mutations in the D‐loop control region (29 mutations in 9 patients) exhibited the highest VAF (median of 56%), reflecting a high prevalence of homoplasmic mutations in this region.

Most substitutions were transitions, with G:C>A:T (39%) and T:A>C:G (23%) being the most frequent mutation types (Figure 1E), consistent with DNA polymerase gamma (Polγ) replication errors.^25^, ^28^ Within coding regions, we detected 105 non‐synonymous and 57 synonymous somatic mutations. *MT‐ND1* (*NADH dehydrogenase 1*, Complex I) exhibited 11 non‐synonymous and no synonymous mutations, representing a significant enrichment of non‐synonymous over synonymous mutations relative to the rest of the mitochondrial coding genome (length‐normalized Fisher's exact test; P = 0.001, P_adj_ = 0.025) (Figure 1F). *MT‐CO1* and *MT‐CO3* (*cytochrome c oxidase subunits 1 and 3*, Complex IV) also showed a skewed non‐synonymous to synonymous ratio (17 vs. 2 P = 0.03, P_adj_ = 0.38 for *MT‐CO1*; and 11 vs. 1 P = 0.008, P_adj_ = 0.12 for *MT‐CO3*). In contrast, mutations in *ATP6* (*n* = 5) and *ATP8* (*n* = 1) (*ATP synthase membrane subunits 6 and 8*, Complex V) were all synonymous, consistent with other reports suggesting negative selection against amino acid–altering variants in ATP synthase.^30^ This potentially non‐random distribution suggests functional effects, though validation in a larger CML cohort is needed due to multiple comparison limitations.

### High mutational burden and homoplasmic mtDNA mutations are enriched in CML patients with favorable response to **imatinib**

To investigate whether the observed mtDNA mutational landscape influences treatment outcomes, we focused on a subset of 79 imatinib‐treated patients from the ALLG CML6^37^ and CML9^38^ clinical trials (Supporting Information S2: Table 9). Patients were selected to include approximately equal proportions of favorable responders (Group A, *n* = 43) and suboptimal responders (Group B, *n* = 33), and 3 patients classified as treatment failure/progression (Group C). Given the limited sample size of Group C, statistical comparisons were restricted to groups A and B, with Group C included for descriptive purposes only.

Group C harbored four heteroplasmic somatic mtDNA mutations across three patients (0, 1, and 3 mutations per patient), with a median VAF of 4.8% (range 4.6–5.4%) and no homoplasmic mutations. Mutations were located in Complex I genes (*MT‐ND1* and *MT‐ND5*) in patient #P0074, while patient #P0175 carried a single variant in *MT‐RNR1* (m.1201A>G).

**Figure 2:**
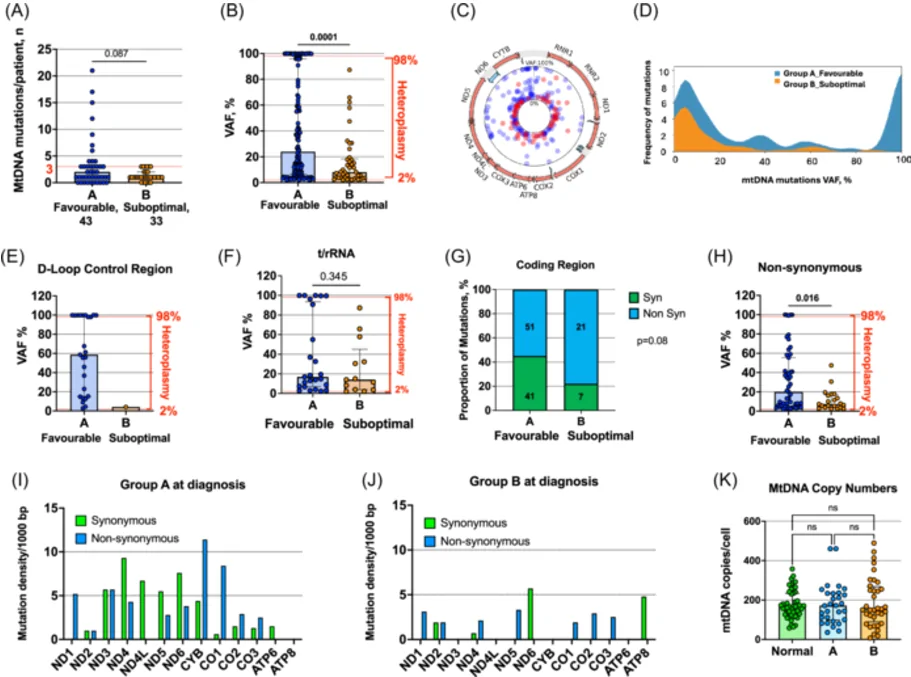
**Association between mitochondrial DNA (mtDNA) mutations and response to imatinib (IM)**. **(A)** Mutational burden in 43 chronic myeloid leukemia (CML) patients with favorable response to imatinib (Group A, blue) and 33 patients with suboptimal responses (Group B, orange). Each dot represents the number of mtDNA mutations in each patient (Mann–Whitney *U* test, median with interquartile range). **(B)** Variant allele frequencies (VAFs) of mtDNA mutations in Group A and B patients (Mann–Whitney test, median with interquartile range). **(C)** Circos plot representing the landscape of mtDNA mutations in Group A (blue) and B (red) patients. The dots represent the VAF distribution from 2% (center) to 100%, illustrating the prevalence and clonal expansion of mutations in each group. **(D)** Distribution of VAFs of mtDNA mutations in Group A (blue) and Group B (orange) patients. The *x*‐axis shows mtDNA mutation VAF (%) ranging from 2% to 100%, and the *y*‐axis shows the number of mutations (%). **(E)** VAFs of mtDNA mutations in the D‐loop control region and **(F)** mitochondrial transfer RNA and ribosomal RNA (t/rRNA) genes in Group A and B patients (Mann–Whitney *U* test, median with interquartile range). **(G)** Proportion of synonymous (green) and non‐synonymous (blue) mutations (Fisher's exact test). **(H)** VAFs of non‐synonymous mutations in Group A and B patients (Mann–Whitney *U* test, median with interquartile range). **(I, J)** Distribution of synonymous (green) and non‐synonymous (blue) mtDNA mutations in the coding region. The number of mutations/1000 bp was normalized on the gene size. Distribution in Group A (I) and Group B (J) patients. **(K)** Boxplot of mtDNA copy number per cell in normal (*n* = 47, green), Group A (*n* = 30, blue), and Group B (*n* = 36, orange). Data were calculated as the ratio of mtDNA (*MT‐ND1* and *MT‐CYB*)/nDNA (*GUSB*) by polymerase chain reaction (PCR) (Kruskal–Wallis test, mean with SEM). ns, non‐significant P > 0.1.

Somatic mtDNA point mutations were detected in both Group A and B patients (77% vs. 82%, respectively). However, Group A showed a higher mutational burden, with 142 mutations (median 2 per patient, range 0−21) compared with 42 mutations in Group B (median 1 per patient, range 0−3) (Figure 2A), and significantly higher VAFs (median 24% vs. 8.1%; P = 0.0001) (Figure 2B). All 29 homoplasmic mutations were restricted to Group A, whereas Group B harbored exclusively heteroplasmic mutations, the majority of which exhibited low VAF (<20%) (Figure 2C,D). An exploratory receiver operating characteristic analysis for achievement of MMR at 12 months (area under the curve = 0.72, 95% CI 0.59–0.85; P = 0.004) identified an optimal cutoff of 2.5 mutations, which was rounded to ≥3 for biological interpretability.

Distribution analysis revealed a hotspot in the D‐loop in Group A patients (25 variants, median VAF 58% vs. 1 variant, VAF 4.1% in IM‐Res; Figure 2E), while structural RNA regions showed no selective differences (Figure 2F). Group A patients further exhibited a balanced ratio of synonymous and non‐synonymous mutations, whereas Group B patients had a skewed enrichment of non‐synonymous mutations at lower VAF (Figure 2G,H).

Complex‐level analysis showed that in Group A patients, synonymous mutations were significantly enriched in Complex I, compared to the rest of the coding genome (32 vs. 9; P_adj_ = 0.017) (Figure 2I). In contrast, non‐synonymous mutations were more broadly distributed across respiratory chain complexes, with a relative enrichment observed in Complex III (cytochrome b, *MT‐CYB*, P_adj_ = 0.005), and *MT‐CO1* (Complex IV) showing enrichment of non‐synonymous over synonymous variants (13 vs. 1; P_adj_ = 0.028) (Figure 2I). In Group B patients, mutations were broadly distributed across the genome. Predicted pathogenicity of non‐synonymous mutations, assessed by PolyPhen and SIFT, was comparable between groups (Supporting Information S1: Figure 1A,B).

We next investigated whether the observed mutational differences between the two groups were associated with changes in mtDNA abundance in the bulk TWCs (Figure 2K). No significant differences were observed in mtDNA/nDNA ratios as determined by Q‐PCR (median mtDNA copies/cell 171 vs. 152 in IM‐S and IM‐Res), suggesting that differences in mutational burden are not driven by mtDNA content.

Together, these data suggest that Group A and Group B patients differ in mtDNA mutational burden and genomic distribution, highlighting potential functional consequences for mitochondrial activity.

### LSPCs from patients with suboptimal molecular response exhibit increased mitochondrial respiration and content

To investigate metabolic differences underlying TKI response, we measured the bioenergetic profile from a subset of 12 CP‐CML patients with CD34^+^ LSPCs available at the time of diagnosis (Supporting Information S2: Table 2). Group A samples included in these functional assays carried a higher mtDNA mutational burden (median 2 mutations/patient, range 1–12) with significantly higher VAF (median 56%, range 2.5–97.1%) compared with Group B patients (median 1 mutation/patient, range 1–2; median VAF 5.8%, range 2.1–12.5%; P = 0.0002; Supporting Information S2: Table 9).

**Figure 3:**
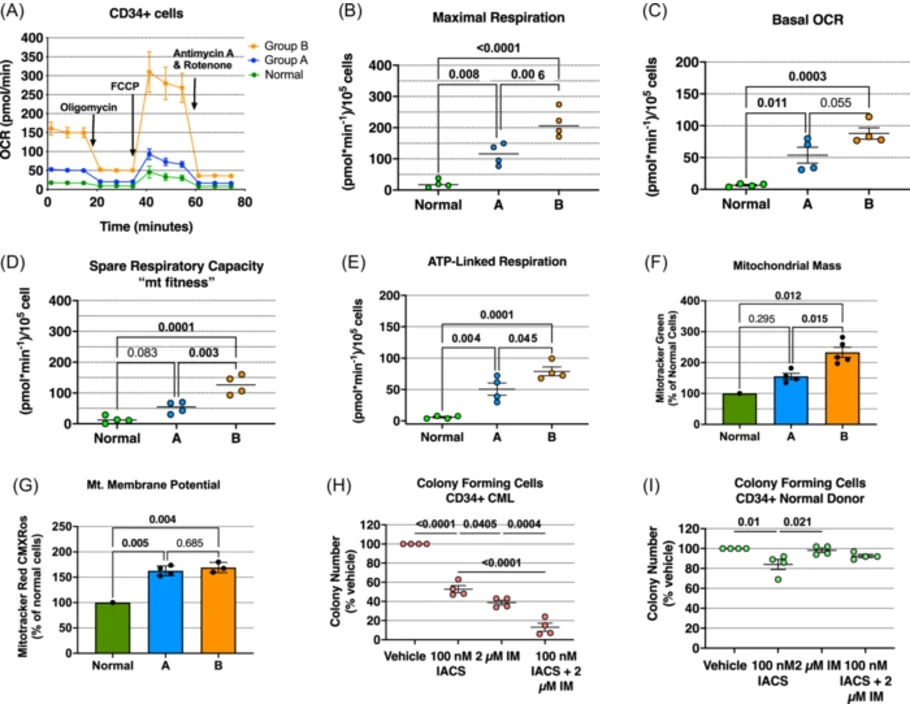
**Mitochondrial function in CD34^+^ cells. (A)** Representative respirometry output in CD34^+^ leukemic stem and progenitor cells (LSPCs) (mean with SEM). **(B)** Maximal respiration after injection of carbonyl cyanide‐4 (trifluoromethoxy) phenylhydrazone (FCCP), **(C)** basal oxygen consumption rate (OCR), **(D)** spare respiratory capacity (measured as difference of maximal respiration and basal respiration), and **(E)** ATP‐linked respiration (after injection of oligomycin, which inhibits the mitochondrial ATP synthase, or Complex V) of four normal hematopoietic stem and progenitor cells (HSPCs) (green), four Group A (blue) and four Group B CD34^+^ LSPCs (red) at diagnosis (ordinary one‐way analysis of variance [ANOVA], mean with SEM). **(F)** Mitochondrial content was assessed by the median fluorescence intensity (MFI) of Mitotracker Green‐labeled CD34^+^ CML cells (four Group A, five Group B), normalized to that of normal control cells (seven healthy donors) (ordinary one‐way ANOVA, mean with SEM); **(G)** Membrane potential was measure by the MFI of MitoTracker Red CMXRos‐labeled CD34^+^ CML cells (four Group A and three Group B), normalized to that of normal control cells (five healthy donors) (ordinary one‐way ANOVA, mean with SEM). **(H)** Colony numbers following 3 days of drug treatment of CD34^+^ LSPCs (four CML patients; three Group B [#P0175*, #NIL003, #P0108] and one Group A [*#NIL002*]. *#P0175 progressed to central nervous system lymphoid blast crisis at 9 months and deceased after about 2.5 years after diagnosis) (ordinary one‐way ANOVA, mean with SEM). **(I)** Colony numbers following 3 days of drug treatment of CD34^+^ HSPCs (four healthy controls) (ordinary one‐way ANOVA, Mean with SEM). All CD34^+^ cell samples used for the functional assays, the experiments performed with each sample, and the corresponding clinical data are provided in Supporting Information S2: Table 2; mtDNA mutation status is reported in Supporting Information S2: Table 10. Mt, mitochondria; OCR, oxygen consumption rate.

Mitochondrial respiration was assessed using the Seahorse Mito Stress Test. Group B LSPCs exhibited higher maximal respiration compared with Group A (mean 206 vs. 115 pmol/min/10^5^ cells; P = 0.006), with a trend toward increased basal OCR (mean 80.3 vs. 54 pmol/min/10^5^ cells; P = 0.055). Both basal and maximal respiration were also higher in CML LSPCs compared with normal CD34^+^ HSPCs (Figure 3A–C). Spare respiratory capacity, reflecting the ability of mitochondria to respond to energetic stress, and ATP‐linked respiration, a measure of ATP‐generating capacity, were further increased in Group B LSPCs compared with Group A and normal CD34^+^ HSPCs (Figure 3D,E).

We next examined whether these functional differences were associated with mitochondrial abundance or polarization by measuring mitochondrial mass and membrane potential by flow cytometry. MitoTracker Green staining revealed a 1.5‐fold increase in mitochondrial content in Group B compared with Group A LSPCs (P = 0.015; Figure 3F, Supporting Information S1: Figure 2), whereas mitochondrial membrane potential measured by MitoTracker RedCMXRos was similar between groups (Figure 3G). Together, these data reveal differences in mitochondrial respiration and mitochondrial content among LSPCs from patients with different molecular response categories.

We next treated LSPCs with the Complex I inhibitor IACS‐01075,^43^ which pharmacologically phenocopies reduced ETC activity caused by mutations in multiple mitochondrial protein‐coding genes. Low‐dose IACS‐010 759 (100 nM) reduced colony formation by 47%, while imatinib alone caused ~61% reduction. Combined treatment nearly abolished colonies (87% reduction; Figure 3H). Normal CD34^+^ HSPC colonies were not affected by the combination therapy and showed a mean reduction of 16% when treated with IACS‐010 759 alone (P = 0.01, Figure 3I).

### Metabolic reprogramming and mtDNA variant enrichment distinguish LSPCs in CML patients with favorable versus suboptimal imatinib response

**Figure 4:**
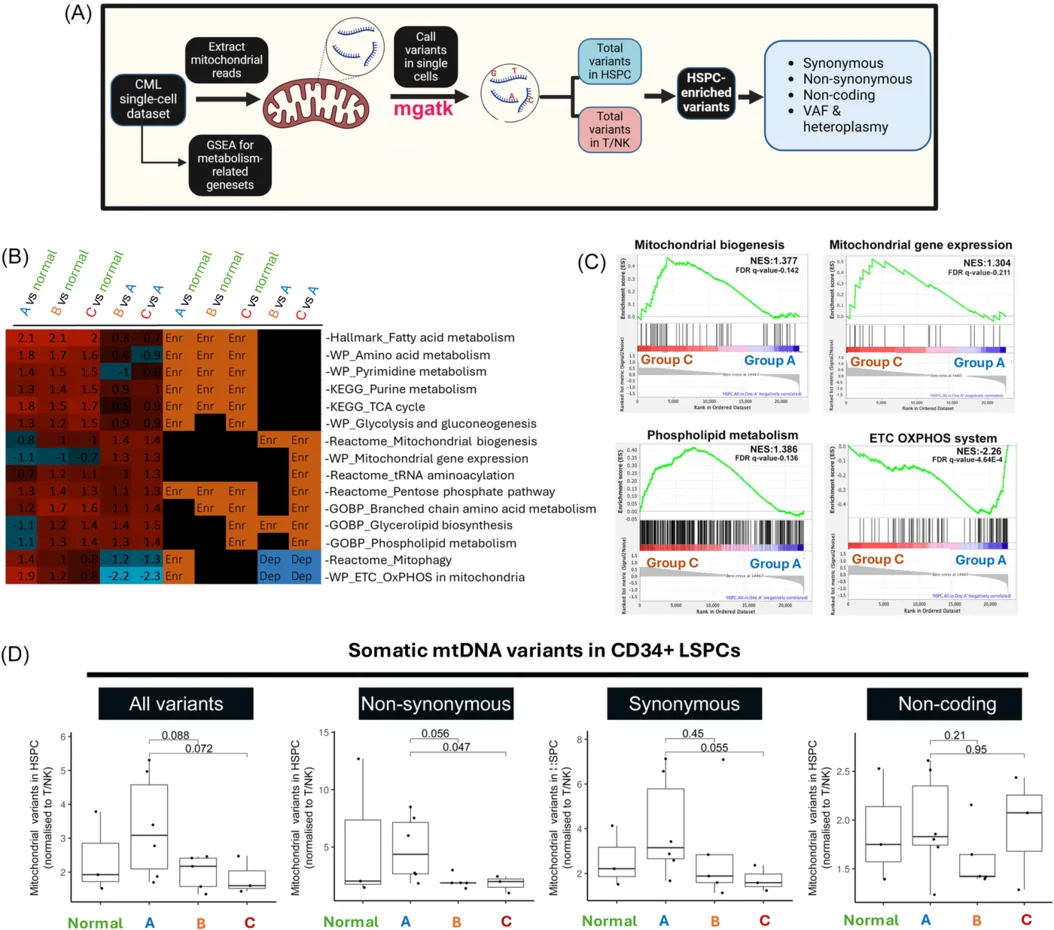
**Single‐cell transcriptomics analysis.** We maintained the original classification according to Krishnanet al.,^13^ as described in their paper. Chronic myeloid leukemia (CML) patients in chronic phase (CP) from the Singapore General Hospital (*n* = 24) were categorized as follows: optimal responders to imatinib (Group A; *n* = 9, blue); suboptimal responders to imatinib but with favorable responses to second‐ or third‐line tyrosine kinase inhibitors (TKIs) (Group B; *n* = 9, orange); and treatment failures with pan‐TKI resistance and progression to blast crisis (Group C; *n* = 6, Red), according to European LeukemiaNet criteria.^34^ Diagnostic bone marrow samples were collected prior to TKI therapy. Normal bone marrow mononuclear cells (*n* = 5, green; donors aged 36–47 years) were obtained from healthy individuals through commercial sources (StemCell Technologies).^12^ **(A)** Schematic overview of the analysis pipeline used to extract and annotate mitochondrial DNA (mtDNA) variants from single‐cell 10 × 5′ single‐cell RNA sequencing (scRNA‐seq) data, alongside gene‐set enrichment analysis (GSEA). **(B)** Heatmap displays normalized enrichment scores (NES) for curated metabolic gene sets. Enrichment was assessed for leukemic stem and progenitor cells (LSPCs) from Group A, B, and C patients (*n* = 24 total) relative to healthy donor CD34^+^ cells (*n* = 5; *control, Ctrl*), and for direct comparisons between LSPC subgroups. Statistical significance (FDR ≤ 0.25) is indicated as enriched (Enr) or depleted (Dep). **(C)** GSEA comparing LSPCs from Group C and Group B patients reveals differential enrichment of mitochondrial and metabolic pathways, including “Reactome_ mitochondrial biogenesis,” “WP_Mitochondrial gene expression,” and “GOBP_Phospholipid metabolism,” alongside downregulation of “WP_ETC_OXPHOS in mitochondria” gene set. **(D)** Relative mtDNA variant burden in LSPCs, normalized to T and NK cells as internal controls (6 Group A, 5 Group B, and 3 Group C patients; 3 normal). Statistical significance was assessed using a two‐sided *t*‐test (P < 0.05). Mitochondrial gene‐set analyses in the 5′ scRNA‐seq dataset (ID: EGAS00001005509). ETC, electron transport chain; FDR, false discovery rate; HSPC, hematopoietic stem and progenitor cell; OXPHOS, oxidative phosphorylation; VAF, variant allele frequency.

To investigate transcriptional programs associated with metabolic remodeling in LSPCs, we analyzed the published 10 × 5′ scRNA‐seq dataset generated by Krishnan et al.^13^ of diagnostic bone marrow CD34^+^ cells from 24 CP‐CML patients and 8 healthy donors (see the 2 section). We performed GSEA against a curated panel of metabolic and mitochondrial pathways (Supporting Information S2: Table 5) and extracted mitochondrial reads for sc‐mtDNA variant analysis (Figure 4A).

Compared with normal HSPCs, LSPCs showed enrichment of pathways related to fatty acid biosynthesis, amino acid metabolism, nucleotide synthesis, the tricarboxylic acid cycle (TCA), glycolysis/gluconeogenesis, and branched‐chain amino acid (BCAA) catabolism, consistent with previous reports (Figure 4B).^17^, ^53^

Within patient subgroups, Group B displayed enrichment of “Reactome_mitochondrial biogenesis” and “GOPB_glycerolipid biosynthesis” pathways compared with Group A (Figure 4B). Group C similarly exhibited transcriptional remodeling of mitochondrial and lipid metabolism relative to Group A (Figure 4B). Consistent with this, GSEA identified enrichment of “Reactome_mitochondrial biogenesis,” “WP mitochondrial gene expression,” “GOBP phospholipid metabolism,” and “GOPB_glycerolipid biosynthesis” pathways (Supporting Information S1: 3C,D), indicating enhanced lipid production, storage, membrane remodeling, and lipid‐mediated signaling (Figure 4B,C). Anaplerotic pathways, including the pentose phosphate pathway and BCAA catabolism, were also upregulated (Supporting Information S1: 3E,F), suggesting increased production of reduced nicotinamide adenine dinucleotide phosphate (NADPH) and replenishment of TCA cycle intermediates to support metabolic flexibility.

In contrast, Group A LSPCs displayed higher expression of mitophagy‐related genes and components of the ETC OXPHOS machinery, potentially reflecting compensatory adaptation to impaired mitochondrial function.^30^

Single‐cell analysis of mtDNA variants was performed after normalization to T/NK cells within the same scRNA‐seq dataset to control for background variation and technical noise (Figure 4D and Supporting Information S1: Figure 4). Total mtDNA variant burden showed a trend toward higher numbers in Group A compared with Groups B and C (P = 0.088, and P = 0.072, respectively). Notably, the number of non‐synonymous mtDNA variants was significantly higher in Group A compared with Group C (P = 0.047), with a similar trend observed relative to Group B (P = 0.056). Synonymous variants also showed a trend toward higher levels in Group A compared with Group C.

These data are consistent with our bulk mtDNA sequencing results and, in an independent cohort, show that higher numbers of mtDNA mutations and higher VAF are associated with response to imatinib.

### mtDNA mutations in CML patients are associated with better response to imatinib

**Figure 5:**
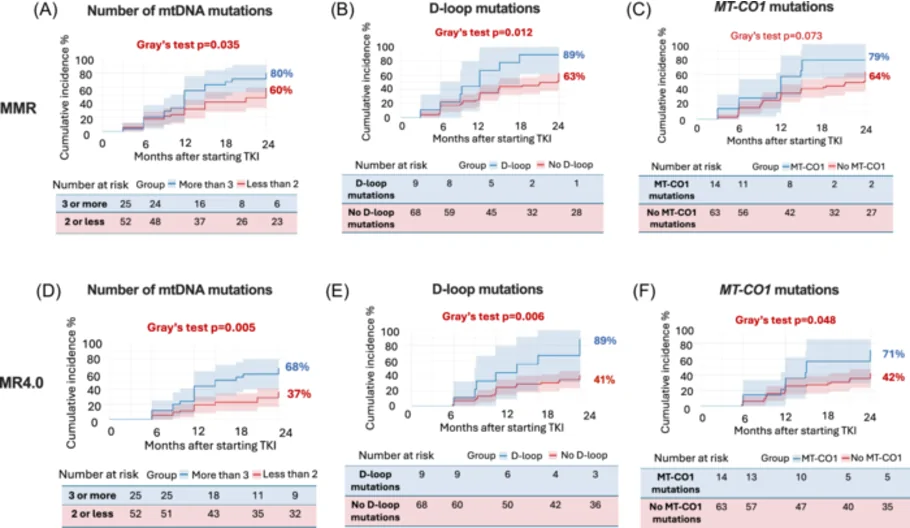
**Impact of mitochondrial DNA (mtDNA) mutations on imatinib response. (A, D)** Cumulative incidence of major molecular response (MMR) and MR4.0 in patients with ≥3 versus ≤2 total mtDNA mutations. (A) MMR: 80% versus 60% (95% CI, 63–97 vs. 46‐73; Gray's test P = 0.035); (D) MR4.0: 68% versus 37% (95% CI 49–87 vs. 23–50; P = 0.005). **(B, E)** Cumulative incidence of MMR and MR4.0 in patients with D‐loop mutations versus non‐D‐loop mutations. (B) MMR: 89% versus 63% (95% CI, 62–100 vs. 52–75; Gray's test P = 0.012); (E) MR4.0: 89% versus 41% (95% CI 64–100 vs. 30–53; P = 0.006). **(C, F)** Cumulative incidence of MMR and MR4.0 in patients with *MT‐CO1* mutations versus non‐*MT‐CO1* mutations. (C) MMR: 79% versus 64% (95% CI, 55–100 vs. 51–76; Gray's test P = 0.073); (F) MR4.0: 71% versus 42% (95% CI 46–97 vs. 30–55; P = 0.048).

We next evaluated the impact of mtDNA mutations on molecular response to imatinib and clinical outcomes, including OS, FFS, and progression to blast crisis. Patients with a higher number of mtDNA mutations, or with D‐loop or *MT‐CO1* variants, achieved superior molecular responses by 24 months. Using a cutoff of ≥3 mutations, patients had higher cumulative incidences of MMR (80% vs. 60%; P = 0.035) and MR4.0 (68% vs. 37%; P = 0.005), compared with patients harboring ≤2 mutations (Figure 5A,D). D‐loop mutations were associated with higher MMR (89% vs. 63%; P = 0.012) and MR4.0 (89% vs. 41%; P = 0.006; Figure 5B,E), and *MT‐CO1* mutations were also associated with higher MR4.0 (71% vs. 42%; P = 0.048) and a trend toward increased MMR (79% vs. 64%; P = 0.073; Figure 5C,F).

Univariate Fine–Gray competing‐risk analyses confirmed that ≥3 mtDNA mutations were consistently associated with MMR and MR4.0. Among clinical variables, shorter *BCR::ABL1* HT^51^ and smaller spleen size were strong predictors of molecular response (Table 1). In multivariate models including HT, palpable spleen length, and mtDNA mutation burden, high mtDNA load remained independently associated with higher MMR and MR4.0 (Table 1). Inclusion of mtDNA mutations improved model performance, as reflected by higher global Wald statistics.

**Table 1:** Fine–Gray competing risks analysis for major molecular response (MMR), MR4.0, and MR4.5.

Univariate, variable	Category/unit	MMR sHR (95% CI)	P‐value	MR4.0 sHR (95% CI)	P‐value
Gender	Male versus female	0.72 (0.41–1.24)	0.229	**0.50 (0.27–0.95)**	**0.035**
Age	Continuous (y)	1.01 (0.99–1.03)	0.27	**1.05 (1.02–1.07)**	**<0.001**
Age	≥50 versus <50	1.01 (0.60–1.71)	0.96	**2.17 (1.13–4.16)**	**0.02**
Sokal score	Medium versus low	0.75 (0.39–1.43)	0.374	1.05 (0.48–2.28)	0.906
Sokal score	High versus low	0.46 (0.21–1.01)	0.054	0.49 (0.17–1.39)	0.179
**Spleen size, cm**	**Continuous**	**0.91 (0.85–0.97)**	**0.004**	**0.77 (0.66–0.88)**	**<0.001**
**HT, days**	**Continuous**	**0.90 (0.85–0.96)**	**<0.001**	**0.80 (0.73–0.87)**	**<0.001**
**mtDNA mutations**	**High (≥3) versus low (<3)**	**1.82 (1.05–3.17)**	**0.033**	**2.74 (1.43–5.22)**	**0.0023**
D‐loop	**Yes versus no**	**2.31 (1.11–4.83)**	**0.025**	**2.97 (1.53–5.74)**	**<0.001**
MT‐CO1	Yes versus no	1.75 (0.88–3.48)	0.11	**2.13 (1.08–4.21)**	**0.029**

	**Category/unit**	**MMR sHR (95% CI)**	**P‐value**	**MR4.0 sHR (95% CI)**	**P‐value**
**Multivariate Model 1**					
**HT, days**	**Continuous (days)**	**0.91 (0.85–0.96)**	**<0.001**	**0.83 (0.75–0.91)**	**<0.001**
**Spleen size, cm**	Continuous (cm)	0.95 (0.89–1.01)	0.11	**0.85 (0.76–0.96)**	**0.011**
**Global Wald test**	**2 df**	**–**	**<0.001; 16.1 statistics**	**–**	**<0.001; 23.4 statistics**
**Multivariate Model 2**					
**mtDNA mutations**	**High (≥3) versus low (<3)**	**2.39 (1.39–4.11)**	**<0.001**	**2.23 (1.11–4.47)**	**0.024**
**HT, days**	**Continuous (days)**	**0.92 (0.87–0.98)**	**0.005**	**0.85 (0.77–0.94)**	**0.001**
**Spleen size, cm**	**Continuous (cm)**	**0.93 (0.87–0.99)**	**0.017**	**0.84 (0.75–0.94)**	**0.002**
**Global Wald test**	**3 df**	**–**	**<0.001; 23.1 statistics**	**–**	**<0.001; 28.1 statistics**

*Note*: Bold font indicates statistically significant results (P < 0.05).

Abbreviations: CI, 95% confidence interval; df, degrees of freedom; HR, subdistribution hazard ratios (sHR > 1 indicates a higher likelihood of achieving MMR, MR4.0 or MR4.5; better outcome); HT, halving time^51^; MMR, major molecular response (*BCR::ABL1* ≤ 0.1% international scale, IS); MR4.0 (*BCR::ABL1* ≤ 0.01% IS); MR4.5 (*BCR::ABL1* ≤ 0.0032% IS); mtDNA, mitochondrial DNA.

Analysis of OS, FFS, and progression to blast crisis did not reach statistical significance, likely due to the limited number of events; however, there was a trend toward higher 2‐year FFS in patients with ≥3 versus ≤2 mtDNA mutations (95% vs. 75%; P = 0.059) (Supporting Information S1: Figure 5).

## Discussion: 942

This study provides the first comprehensive analysis of mtDNA mutations in CML and identifies a link between mtDNA mutational burden and response to imatinib therapy. Somatic mtDNA mutations were highly prevalent at diagnosis, with 77% of patients carrying at least one mutation, and the mutational landscape differed significantly between patients who achieved optimal molecular responses and those with suboptimal response to imatinib. Patients achieving MMR and deeper remissions harbor a higher number of mtDNA mutations, frequently at higher VAF, whereas patients with suboptimal response exhibit fewer mutations, predominantly heteroplasmic and of low VAF. These findings further support an association between mtDNA mutational burden and therapeutic outcome, highlighting mtDNA profiling as a potential stratification approach in CML.

Context‐dependent associations between mtDNA mutational burden, metabolic remodeling, and therapy outcomes have been reported in solid tumors, including colorectal, melanoma, and head‐and‐neck cancers, supporting the potential clinical relevance of mtDNA variation as a stratification marker.^23^, ^25^, ^26^, ^30^, ^54^, ^55^ In prostate cancer, non‐synonymous mtDNA mutations in Complex I have been associated with adverse outcome, OXPHOS remodeling, and altered expression of metabolic enzymes involved in pyruvate and succinate metabolism.^56^ Cybrid models using patient‐derived renal carcinoma cells have demonstrated that mutations in *MT‐CYB* induce a metabolic shift toward reductive carboxylation of glutamine to support lipid synthesis and proliferation of cancer cells.^34^ D‐loop mutations correlate with lower mtDNA copy number, poorer chemotherapy response, and reduced five‐year survival in esophageal cancer.^57^ Conversely, in head and neck squamous cell carcinoma^58^ and oral squamous cell carcinoma,^59^, ^60^ D‐loop mutations were associated with better disease‐specific survival.

In this framework, the observed association between mtDNA mutational burden and response to imatinib may reflect differences in mitochondrial adaptation under energetic stress, with individual pathogenetic variants potentially contributing to these phenotypes in a context‐dependent manner. Single‐cell transcriptomic profiling showed differential expression of mitochondrial pathways, including ETC components and mitophagy‐related genes, alongside with altered mitochondrial biogenesis programs between response groups. These transcriptomic differences are consistent with variation in mitochondrial state and cellular energy programs under therapeutic pressure.^30^, ^61^

Consistent with this model, cells from patients with suboptimal response to imatinib exhibited lower mtDNA mutational burden and predominantly low‐VAF heteroplasmic variants. Transcriptomic analyses demonstrated upregulation of mitochondrial biogenesis, RNA processing, and glycerolipid biosynthesis in these patients, likely reflect increased metabolic capacity, supporting maintenance of mitochondrial function and cellular energy demand.^16^, ^62^ Functional assays further revealed that leukemic progenitor cells from patients with suboptimal response to TKI maintain a metabolically adaptable mitochondrial network capable of enhanced OXPHOS. Although limited by a small sample size, these functional data provide preliminary evidence of differences in mitochondrial respiratory activity between response groups, which warrants further study as a potential therapeutic vulnerability.

Pharmacologic OXPHOS inhibition with the Complex I inhibitor IACS‐010759, reduced mitochondrial function and clonogenic potential in LSPC, enhancing their susceptibility to TKI therapy. This approach functionally models impaired ETC activity arising from disruption of mitochondrial respiration, including alterations that may occur downstream of mtDNA mutations, since any impairment of the ETC reduces ATP production and creates a metabolic bottleneck.^63^ Due to the clinical toxicity profile of IACS‐010759^64^ future strategies may focus on selective delivery systems, alternative mitochondrial targets with improved safety profiles, or transient inhibition approaches to limit off‐target toxicity. To address this, a recent drug‐repurposing screen identified lomerizine, a calcium channel blocker, as a selective mitochondrial metabolism inhibitor in CML, with reduced systemic toxicity.^65^ These findings support further investigation of metabolism‐informed therapeutic strategies.

Several limitations warrant consideration. Although our findings were independently validated in the Singapore cohort, the sample sizes in both cohorts remain modest, which limits the statistical power of subgroup analyses. mtDNA mutational burden is associated with molecular response, but the specific number of mutations that defines a low burden would require validation in larger and independent cohorts of CML patients. Finally, bulk leukocyte sequencing cannot fully resolve cell‐type–specific contributions, and single‐cell 5′ 10x Genomics detection of mtDNA is constrained by uneven coverage, low mitochondrial read depth, and reliance on mtRNA transcripts, potentially underestimating heteroplasmy and low‐frequency variants.^48^ Future studies could address these challenges by integrating mtDNA variant analysis into existing bulk or single‐cell datasets and employing targeted single‐cell mtDNA sequencing approaches,^48^, ^66^ which would allow more accurate detection of heteroplasmic variants and cell‐type–specific contributions.

In summary, our study highlights mitochondrial genetic variation as a potential biomarker in CML, revealing difference in metabolic states between patient groups. Collectively, these findings support a model in which mtDNA mutational architecture reflects distinct mitochondrial and metabolic states associated with therapeutic fitness in CML. Integrating mitochondrial genomics with metabolic profiling may therefore improve patient stratification and uncover targetable vulnerabilities relevant to TKI resistance. These insights may extend to other malignancies treated with targeted agents, providing a framework for precision oncology interventions that exploit metabolic vulnerabilities.

## AUTHOR CONTRIBUTIONS

**Ilaria S. Pagani**: Conceptualization; investigation; funding acquisition; writing—original draft; methodology; writing—review and editing; formal analysis; project administration; data curation; resources; validation; visualization; supervision. **Vaidehi Krishnan**: Investigation; writing—review and editing; formal analysis; data curation; validation; methodology; visualization. **John F. Ouyang**: Investigation; writing—review and editing; methodology; software; formal analysis; data curation; visualization. **Chung H. Kok**: Writing—review and editing; methodology; formal analysis. **BeiJun Chen**: Formal analysis; software. **Kian Leong Lee**: Formal analysis; software; writing—review and editing. **Cong M. Pham**: Software; formal analysis. **Paul Wang**: Software; formal analysis; writing—review and editing. **Elyse Page**: Investigation. **Phuong Dang**: Investigation. **Verity A. Saunders**: Investigation. **Jane James**: Investigation. **Kelly Lim**: Investigation. **Stefano Mangiola**: Validation; formal analysis. **Yingnan Gao**: Validation; formal analysis. **Naranie Shanmuganathan**: Writing—review and editing. **Agnes Yong**: Writing—review and editing. **Susan Branford**: Writing—review and editing. **Charles Chuah**: Writing—review and editing. **David T. Yeung**: Writing—review and editing. **Deborah L. White**: Writing—review and editing. **Vignir G. Helgason**: Writing—review and editing; methodology. **Daniel Thomas**: Writing—review and editing; methodology. **Sin Tiong Ong**: Writing—review and editing; supervision; funding acquisition. **Timothy P. Hughes**: Writing—review and editing; supervision; funding acquisition. **David M. Ross**: Writing—review and editing; supervision; conceptualization; funding acquisition.

## CONFLICT OF INTEREST STATEMENT

The authors declare no conflicts of interest.

## ETHICS STATEMENT

Human primary blood samples, including samples from CML patients and healthy donors, were obtained with written informed consent in accordance with the Declaration of Helsinki, and with approval from the Central Adelaide Local Health Network Human Research Ethics Committee (HREC/14/RAH/316).

## FUNDING

This research project was supported by SAHMRI Mid‐Career Seed Funding Grant (I.S.P.); Cancer Council SA Research Mid‐Career Research Fellowship on behalf of its donors and the State Government of South Australia through the Department of Health and Wellbeing (I.S.P.); Cancer Council SA Research COVID‐19 Hardship Grant (I.S.P.); Investigator Grant # 2007 908 (T.P.H.); and Contributing Haematologists Committee Research Grant (D.M.R., I.S.P., and D.L.W.). National Medical Research Council Singapore: CIRG/1468/2017, MOH‐000 602, MOH‐000 059, and CIRG16nov032 (S.T.O.). Leukemia & Lymphoma Synergistic Team Award with support from the Mike & Sofia Segal Foundation (D.T.), NHMRC Ideas Grants (D.T.), and The Medical Research Future Fund (D.T.). Open access publishing facilitated by Adelaide University, as part of the Wiley ‐ Adelaide University agreement via the Council of Australasian University Librarians.

## Supporting information

Supporting Information.

Supporting Information.

## Data Availability

The data that support the findings of this study are openly available in single‐cell transcriptomics of human chronic myeloid leukemia at https://ega-archive.org/studies/EGAS00001005509, reference number EGAS00001005509. We declare that data supporting the findings of this study are available within this manuscript and its supplementary information files.
