# Supplementary material for: Mitochondrial DNA mutations are associated with impaired oxidative phosphorylation and enhanced imatinib response in chronic myeloid leukemia
Source: Hemasphere. 2026 Sep 7;10(9):e70429. doi: 10.1002/hem3.70429 (PMC13549372; doi:10.1002/hem3.70429)
Supplement: Supplementary file 1 [file HEM3-10-e70429-s002.docx]

Supplementary Materials for

**Mitochondrial DNA Mutations Are Associated with Impaired Oxidative Phosphorylation and Enhanced Tyrosine Kinase Inhibitor Response in Chronic Myeloid Leukemia**

**Short Title**

**Mitochondrial DNA Mutations in Chronic Myeloid Leukemia**

**This PDF file includes:**

Materials and Methods

Figures. S1 to S5

Captions for Data S1 to S5

**Other Supplementary Materials for this manuscript include the following:**

Supplementary Tables S1 to S10

S1: Summary of Baseline Features and Clinical Outcomes for CML Patients Undergoing mtDNA Mutational Profiling (Adelaide Cohort)

S2: Patient Cohort and Samples Included in Functional Experiments (Adelaide cohort)

S3: List of mtDNA Somatic Mutations in TWCs (Adelaide cohort)

S4: Patient Cohort and Samples Included in the scRNA sequencing analysis (Singapore cohort)

S5: List of sc-mtDNA Variants (Singapore cohort)

S6: Validation of TWCs Somatic mtDNA Variants in CD34⁺ Cells Using LoFreq and mtGATK (Adelaide cohort)

S7: Validation of sc-mtDNA variants (RNA) in bulk CD34+ LSPCs and MNCs (DNA) in #P147 (Group C, Singapore cohort)

S8: Detailed Per-Patient Characteristics at Study Entry and Clinical Outcomes in the Adelaide Imatinib Cohort

S9: mtDNA Mutations in CML Samples Used for Functional Analyses

S10: GSEA of Normal and CML CD34+ Stem/Progenitor Cells

Materials and Methods

**Oxygen consumption rate (OCR) measurement**

OCR was measured using the Seahorse XF96 Analyzer (Agilent Technologies, California, United States) with the Seahorse XF Cell Mito Stress Test Kit (Agilent, California, United States). Primary cells were cultured overnight in SFM with physiological growth factors and then resuspended in warm Seahorse XF RPMI medium (pH 7.4), supplemented with 10 mM glucose, 1 mM pyruvate, and 2 mM glutamine (all from Agilent Technologies, California, United States). Eight replicates were performed per sample.

A total of 1.2 × 10⁵ cells (50 μL) were seeded per well of a Seahorse XF cell culture plate pre-coated with 22.4 μg/mL Corning® Cell-Tak™ Cell and Tissue Adhesive (Corning, New York, United States) and centrifuged at 800 rpm for 3 minutes without brakes. After centrifugation, warm complete Seahorse medium was gently added to each well, bringing the final volume to 180 μL. The cells were incubated at 37°C in a non-CO₂ incubator for 1 hour.

The cells were then transferred to the Seahorse analyzer, where OCR was measured at baseline and after subsequent injections of specific inhibitors. The first injection was of the ATP-synthase inhibitor oligomycin (1.5 μM), followed by the mitochondrial uncoupler carbonyl cyanide-4-(trifluoromethoxy)phenylhydrazone (FCCP, 2 μM), and rotenone and antimycin A (0.5 μM), inhibitors of complexes III and II, respectively. OCRs were normalized by cell number. These steps allowed for the measurement of the ATP-linked respiration after the injection of oligomycin, and calculated as the last rate measurement before oligomycin injection minus the minimum rate measurement after oligomycin injection, as well as maximal OCR (the highest rate of oxygen consumption achieved after injection of FCCP normalised on the minimum value after the injection of rotenone and antimycin A indicative of the non-mitochondrial oxygen consumption) and basal OCR (the rate of oxygen consumption under normal conditions, normalised on the non-mitochondrial oxygen consumption). The difference between maximal and basal OCR was used to calculate mitochondrial fitness, providing insights into mitochondrial function and capacity.

**Mitochondrial content and membrane potential**

Mitochondrial content and membrane potential were assessed using MitoTracker Green FM and MitoTracker Red CMXRos (Invitrogen Carlsbad, California) fluorescent probes, following the manufacturers’ instructions.

Briefly, 1.2 × 10⁵ cells were washed with cold 1× PBS and pelleted at 1200 rpm for 5 minutes. Cells were resuspended in pre-warmed (37 °C) 1× PBS supplemented with 2% FCS and transferred to a 96-well round-bottom plate. Cells were then incubated for 30 minutes at 37 °C in a humidified incubator with 5% CO₂, protected from light, with anti-human CD34–APC (clone 8G12, BD Biosciences, New Jersey, United States; 1:100), anti-human CD38–PE-Cy7 (clone HIT2, BD Biosciences; 1:100), MitoTracker Green FM (50 nM, Invitrogen Carlsbad, California), and MitoTracker Red CMXRos (5 nM, Invitrogen). DAPI (Sigma-Aldrich, Missouri, United States) was then added at a final concentration of 0.05 ug/mL, and cells were incubated for an additional 1 minute. Following staining, cells were washed with pre-warmed 1× PBS containing 2% FCS and pelleted at 1200 rpm for 5 minutes. The supernatant was discarded, and cells were resuspended in pre-warmed 1× PBS with 2% FCS. Fluorescence was acquired using a BD LSRFortessa X-20 Cell Analyzer, and data were analysed using FlowJo software v10.10.

**Next-generation sequencing of the entire mitochondrial genome**

MtDNA mutations were identified as previously described.^1^ Briefly, genomic DNA was extracted using a phenol/chloroform method. The full 16.9 kb mitochondrial genome was amplified by long-range PCR using overlapping primer sets, and pooled amplicons were subjected to library preparation using the NexteraXT kit (Illumina, San Diego, CA)) and sequenced on an Illumina MiSeq platform.

The full 16.9 kb mtDNA was isolated by long-range PCR, generating two or three overlapping fragments. by using the primers: hmt F1 5'- AACCAAACCCCAAAGACACC-3', hmt R1 5'- GCCAATAATGACGTGAAGTCC-3' (amplicon length 9,289 bp), hmt F2 5'- TCCCACTCCTAAACACATCC-3', hmt R2 5'-TTTATGGGGTGATGTGAGCC-3' (7,626 bp);

or Mito1 F 5'- ACATAGCACATTACAGTCAAATCCCTTCTCGTCCC-3', Mito1 R 5'-TGAGATTGTTTGGGCTACTGCTCGCAGTGC-3' (3,968 bp), Mito2 F 5'-TACTCAATCCTCTGATCAGGGTGAGCATCAAACTC-3', Mito2 R 5'- GCTTGGATTAAGGCGACAGCGATTTCTAGGATAGT-3' (5,513 bp), Mito3 F 5'- TCATTTTTATTGCCACAACTAACCTCCTCGGACTC-3', Mito3 R 5'-CGTGATGTCTTATTTAAGGGGAACGTGTGGGCTAT-3' (7,814 bp).^1,2^

The reaction mix contained 2.5 U Takara LA Taq (Clontech Laboratories, Mountain View, CA), 1X Takara LA PCR Buffer ll (Mg^2+^plus), 0.2 mM dNTPs, 400 nM primers forward and reverse primers and 20 ng of human genomic DNA. PCR was performed using the following conditions: 95°C for two minutes; 30 cycles of 95°C for 15 sec and 68°C for 10 minutes; 68°C for 20 minutes. After PCR, the amplicons were quality checked on agarose gel electrophoresis, purified by either ExoSAP*-*IT (Affimetrix) or [QIAquick Gel Extraction Kit](https://www.qiagen.com/shop/sample-technologies/dna/dna-clean-up/qiaquick-gel-extraction-kit/) (Qiagen), quantified by Qubit*® 3.0* Fluorometer (Thermofisher), and equimolar pooled. Pooled amplicons (1 ng) were used for the library preparation by NexteraXT kit (Illumina, San Diego, CA) following the manufacturer instructions, and ran on Illumina MiSeq by using the Miseq Reagent v3 600 cycle kit.

The figure is generated with *Biorender.com*.

**MtDNA copy number quantification**

Relative mtDNA copy number was quantified by real-time quantitative PCR (qPCR) using two mitochondrial gene targets, *mitochondrially encoded NADH dehydrogenase 1* ***(MT-ND1***; Hs02596873-s1, FAM-MGB) and *mitochondrially encoded cytochrome b* (***MT-CYB;*** Hs02596867-s1, FAM-MGB), normalized to the nuclear reference gene *glucuronidase beta* (***GUSB***.^3^ Reactions were performed in triplicate using TaqMan assays on a QuantStudio™ 7 Flex system (Life Technologies, California, USA). All TaqMan® assays were obtained from Life Technologies (California, USA). Custom primers and probes for *GUSB* were: Forward: 5′-ATTTTGCCGATTTCATGACTGA-3′, Reverse: 5′-GACGGGTACGTTATCCCATGAG-3', Probe: FAM-5′-AGTGTAAGTGGCAGTTTG-3′-MGB. Reactions were set up in 96-well Fast Optical Reaction plates containing 12.5 μL of TaqMan® Universal PCR Master Mix (Life Technologies), 400 nM of each primer, 200 nM of the GUSB MGB probe, 20 ng of genomic DNA, and nuclease-free water in a total volume of 25 μL. All reactions were performed in triplicate on a QuantStudio™ 7 Flex Real-Time PCR System (Applied Biosystems). Thermal cycling conditions were as follows: 2 min at 50 °C, 10 min at 95 °C, followed by 45 cycles of 15 s at 95 °C and 1 min at 60 °C. Ct values for **MT-ND1**, **MT-CYB**, and **GUSB** were determined simultaneously within the same run. Cases and matched controls were analysed on the same plate to minimize inter-assay variability. The relative mtDNA copy number per diploid genome was calculated using the formula: MCN=2×2^−ΔCt^, where ΔCt = Ct_(*MT-ND1* or *MT-CYB*) – Ct_(*GUSB*). The final MCN for each sample was determined by averaging the MCN values for both *MT-ND1* and *MT-CYB*: Final MCN=( MCN*_MT−ND1_*+MCN*_MT−CYB_*/2.

**Calling mtDNA variants from scRNA-seq data**

To identify mtDNA variants at the single-cell level, we analyzed our previously published single-cell RNA sequencing (scRNA-seq) dataset^4^ using the Mitochondrial Genome Analysis Toolkit (mgatk).^5^ Patients details (Singapore cohort) are provided in **Supplementary Table 4**. mgatk was applied to aligned BAM files generated from 10x Genomics data to detect mtDNA variants and quantify allele frequencies across individual cells (pipeline details are provided in the **Supplementary Methods**). The resulting objects were imported into the Signac package (v1.14)^6^ for downstream analysis. Variant coverage was determined using the IdentifyVariants function, retaining variants detected with high confidence in at least two cells (n_cells_conf_detected ≥2). Single-cell allele frequencies were calculated using the AlleleFreq function, and cells were considered to harbor a variant if the allele frequency exceeded 0.1. The median sequencing coverage per cell was 17.7 reads, resulting in a total of 957,796 mtDNA-specific reads mapped across all cells (**Supplementary Figure 4A**).

Using this approach, we identified and annotated mtDNA variants across 54,041 bone marrow cells, facilitating comprehensive analysis of variant prevalence and heterogeneity among patient groups. To assess lineage-specific variant enrichment, we compared the number of mtDNA variants detected in hematopoietic stem and progenitor cells (HSPCs) with those observed in T and NK cells. Because sequencing depth can influence variant detection, the number of variants in HSPCs was normalized to that in T&NK cells by calculating an HSPC-to-T&NK variant ratio for each patient. Variants with VAF >90% in T&NK cells were considered likely germline and excluded from the analysis. The HSPC-to-T&NK variant ratio was calculated for each patient and compared across clinical groups (e.g., imatinib-sensitive vs imatinib-resistant). Using this normalization, we identified a total of 429 mtDNA variants across 41,290 LSPCs, with a median of 96 variants per patient (**Supplementary Table 5)**. Variants were further classified as synonymous, non-synonymous, or non-coding, and category-specific ratios were calculated. For visualization of cell populations in which mtDNA variants were assessed, uniform manifold approximation and projection (UMAP) embeddings were generated from the top 50 principal components of highly variable genes using Seurat (v5.1)^7^ workflow.

**mgatk pipeline details.** mgatk was executed using the ***tenx*** command to process 10x Genomics single-cell data. The aligned input BAM file ("possorted_genome_bam.bam") was specified using the -i flag. The output label and directory were set using the '-n' and '-o' flags, respectively. For accurate cell and molecule identification, we set the unique molecular identifier (UMI) tag to 'UB' using the '-ub' flag, and the barcode tag to 'CB' using the '-bt' flag. The path to the barcodes file was provided using the '-b' flag, pointing to the 'barcodes.tsv' file in each sample's directory, corresponding to the cell barcodes of cells passing QC and present in the final dataset. Finally, we specified the reference genome as GRCh38 using the '-g' flag. The output signac[c] rds object from running the mgatk pipeline was then used for downstream analysis using the signac package (v1.14).

**Validation of mtDNA variants between total leukocytes and matched CD34⁺ LSPCs, and between bulk sequencing and scRNA-seq.**

We validated mtDNA mutations identified in total white cells by confirming their presence in matched bulk CD34⁺ LSPCs. We also compared variant calls from 5′ scRNA-seq data to matched targeted bulk mtDNA sequencing. Detailed methodology, variant calling performance metrics, and results are provided in the **Supplementary Methods** and **Supplementary Tables 6 and 7**.

**Using LoFreq and** mgatk^5^ **for variant calling, we validated six heteroplasmic somatic mutations initially detected in total leukocytes from four CP-CML patients at diagnosis but absent in matched germline controls (LoFreq: mean VAF 5.1%; mtGATK: mean 5.2% at diagnosis) (Supplementary Table 6). These mutations were also identified in CD34⁺ cells with comparable VAFs (LoFreq: mean 6.0%; mtGATK: mean 6.9%), supporting the persistence of clonally dominant mtDNA mutations in the LSPC compartment.**

Next, **validated the mtDNA variant calling from** 5′ scRNA-seq data^4^ by comparing them to those obtained from targeted mtDNA sequencing of matched bone marrow CD34⁺ cells and mononuclear cells (MNCs) from the same patient (#P147) using the Illumina MiSeq platform (**Supplementary table 7**). In bulk CD34⁺ cells, a total of 29 homoplasmic mtDNA variants were identified using the LoFreq algorithm, of which 11 had variant allele frequency (VAF) <98%. For scRNA-seq data, we applied a stringent cutoff of ≥2 cells and a minimum single-cell allele frequency (scAF) of 10% (scAF defined as variant-supporting reads divided by total mitochondrial reads per cell) to reduce false positives. Of the 29 validated variants, 13 were correctly identified (true positives), 16 were missed (false negatives), and 2 additional variants, with scAF of 91% and 96%, were detected that were not present in bulk data (false positives); 34 positions were correctly identified as negatives (true negatives). Although all mtDNA variants identified in CD34⁺ bulk DNA were homoplasmic, their corresponding scAFs in scRNA-seq data showed a median of 96% (range: 16–100%), with lower scAF associated with low coverage. This approach achieved a specificity of 94%, precision of 87%, and overall accuracy of 72%. However, sensitivity remained modest at 45%, with a false-negative rate of 55%, likely due to the intrinsic limitations of the 10x Genomics 5′ platform, including low mitochondrial coverage, read dropouts, and the transcript-based nature of the assay, which limits the ability to capture full-length mitochondrial genomes.^5,8^

Figure S1.

**Association between mtDNA mutations and response to imatinib (IM)**

**(A)** Comparison of variant allele frequencies (VAFs) in non-synonymous mutations in Complex I between IM-S and IM-Res patients (Mann-Whitney U test, median with interquartile range)**.**

**(B-C)** Distribution of PolyPhen-2 **(B)** and SIFT **(D)** scores for non-synonymous mutations in IM-S and IM-Res patients (Mann-Whitney test, median with interquartile range). PolyPhen-2 scores >0.908 are classified as "probably damaging," scores between 0.446 and 0.908 as "possibly damaging," and scores <0.446 as "benign". SIFT scores range from 0 to 1, with scores <0.05 considered **deleterious** and scores ≥0.05 considered **tolerated.**

Figure. S2.

**Mitochondrial function in CD34+ cells leukaemic stem/progenitor cells (LSPCs).**

Representative histograms of Mitotracker Green- and Mitotracker red CMRos-labelled normal CD34+ HSPCs (green) and CD34+ LSPCs (TKI-S in blue and TKI-Res in red) (Median Fluorescent Intensity).

Figure. S3.

**Leading-edge pathway analysis of metabolic gene sets in CD34⁺ LSPCs from sc-RNA-seq.**
Mitochondrial and metabolic gene sets significantly enriched by gene set enrichment analysis (GSEA) in the 5' scRNA-seq dataset (*ID: EGAS00001005509*; **Figure 4B** and **Supplementary Table 10**), including **(A)** Reactome_mitochondrial biogenesis, **(B)** WP_Mitochondrial gene expression, **(C)** GOBP_Phospholipid metabolism, **(D)** GOBP_**Glycerolipid biosynthesis, (E) Reactome_Pentose Phosphate Pathway (PPP)** and **(F)** GOBP_**Branched-Chain Amino Acid (BCAA) metabolism** were subjected to leading-edge analysis. Genes contributing to the enrichment of these pathways were compiled into **metagene lists**, and their expression was evaluated per cell using the "AddModuleScore" function. Module scores were **pseudo-bulked per patient** within the CD34⁺ LSPC compartment and visualized as box-and-whisker plots. Patients were classified as IM-S (n = 9), IM-Res with suboptimal imatinib response but favorable second- or third-line TKI response (n = 9), or IM-Fail with progression to blast crisis (n = 6) (**Supplementary Table 4**), as defined by the European LeukemiaNet criteria.^9^ Statistical significance was assessed using a two-sided t-test (p < 0.05).

Figure. S4.

**Mitochondrial variant analysis in the 5' scRNA-seq dataset (ID: EGAS00001005509).**

**(A)** Coverage of scRNA-seq reads across mitochondrial genes for three representative samples: P654 and P677 (CP^MMR^; optimal responders to imatinib), P147 (CP^Fail^; progression to myeloid blast crisis). **(B-E)** Total mitochondrial variants detected in LSPC clusters (HSC, iMKP, MKP, ERP, EOBM, iNeP, NeP, Lyp, Pro-B) and T/NK cell clusters, stratified by variant type (**B**, all variants; **C**, non-synonymous; **D**, synonymous; **E**, non-coding). Data are shown per patient.

Abbreviations: *Hematopoietic stem cell (HSC), Immature megakaryocyte progenitor (iMKP), Megakaryocyte progenitor (MKP), Erythroid progenitor (ERP), Eosinophil-Basophil Mast cell progenitor (EOBM), Immature Neutrophil progenitor (iNeP), Neutrophil progenitor (NeP), Lymphoid progenitor (Lyp), Pro-B (B cell progenitor).*

**C-**

**D-**

**E-**

Figure. S5.

**Impact of mtDNA mutations on imatinib response.**

Failure-free survival (FFS) according to: **(A)** D-Loop mutations vs non-D-loop at 2 years (100% vs 79%; hazard ratio log rank [HR] 0) and **(B)** at 4 years (100% vs 73%; hazard ratio log rank [HR] 0). **(C)** mutations in *MT-CO1* vs non-*MT-CO1* mutations at 2 years (90% vs 80%; hazard ratio log rank [HR] 0.45; 95% CI, 0.09 – 2.03) and **(D)** at 4 years (90% vs 74%; hazard ratio log rank [HR] 0.39; 95% CI, 0.09 – 1.6). **(E)** mutational burden, ≥3 or <3 mutations at 2 years (95% vs 75%; hazard ratio log rank [HR] 20.18; 95% CI, 0.05 – 0.59) and **(F)** at 4 years (82% vs 75%; hazard ratio log rank [HR] 0.54; 95% CI, 0.18 – 1.63).

**References:**

1.	Pagani, I.S., Kok, C.H., Saunders, V.A., Van der Hoek, M.B., Heatley, S.L., Schwarer, A.P., Hahn, C.N., Hughes, T.P., White, D.L., and Ross, D.M. (2017). A Method for Next-Generation Sequencing of Paired Diagnostic and Remission Samples to Detect Mitochondrial DNA Mutations Associated with Leukemia. J Mol Diagn *19*, 711-721. 10.1016/j.jmoldx.2017.05.009.

2.	Dames, S., Chou, L.S., Xiao, Y., Wayman, T., Stocks, J., Singleton, M., Eilbeck, K., and Mao, R. (2013). The development of next-generation sequencing assays for the mitochondrial genome and 108 nuclear genes associated with mitochondrial disorders. J Mol Diagn *15*, 526-534. 10.1016/j.jmoldx.2013.03.005.

3.	Hosnijeh, F.S., Lan, Q., Rothman, N., San Liu, C., Cheng, W.L., Nieters, A., Guldberg, P., Tjonneland, A., Campa, D., Martino, A., et al. (2014). Mitochondrial DNA copy number and future risk of B-cell lymphoma in a nested case-control study in the prospective EPIC cohort. Blood *124*, 530-535. 10.1182/blood-2013-10-532085.

4.	Krishnan, V., Schmidt, F., Nawaz, Z., Venkatesh, P.N., Lee, K.L., Ren, X., Chan, Z.E., Yu, M., Makheja, M., Rayan, N.A., et al. (2023). A single-cell atlas identifies pretreatment features of primary imatinib resistance in chronic myeloid leukemia. Blood *141*, 2738-2755. 10.1182/blood.2022017295.

5.	Lareau, C.A., Ludwig, L.S., Muus, C., Gohil, S.H., Zhao, T., Chiang, Z., Pelka, K., Verboon, J.M., Luo, W., Christian, E., et al. (2021). Massively parallel single-cell mitochondrial DNA genotyping and chromatin profiling. Nat Biotechnol *39*, 451-461. 10.1038/s41587-020-0645-6.

6.	Stuart, T., Srivastava, A., Madad, S., Lareau, C.A., and Satija, R. (2022). Author Correction: Single-cell chromatin state analysis with Signac. Nat Methods *19*, 257. 10.1038/s41592-022-01393-7.

7.	Hao, Y., Stuart, T., Kowalski, M.H., Choudhary, S., Hoffman, P., Hartman, A., Srivastava, A., Molla, G., Madad, S., Fernandez-Granda, C., and Satija, R. (2024). Dictionary learning for integrative, multimodal and scalable single-cell analysis. Nat Biotechnol *42*, 293-304. 10.1038/s41587-023-01767-y.

8.	Ludwig, L.S., Lareau, C.A., Ulirsch, J.C., Christian, E., Muus, C., Li, L.H., Pelka, K., Ge, W., Oren, Y., Brack, A., et al. (2019). Lineage Tracing in Humans Enabled by Mitochondrial Mutations and Single-Cell Genomics. Cell *176*, 1325-1339 e1322. 10.1016/j.cell.2019.01.022.

9.	Apperley, J.F., Milojkovic, D., Cross, N.C.P., Hjorth-Hansen, H., Hochhaus, A., Kantarjian, H., Lipton, J.H., Malhotra, H., Niederwieser, D., Radich, J., et al. (2025). 2025 European LeukemiaNet recommendations for the management of chronic myeloid leukemia. Leukemia. 10.1038/s41375-025-02664-w.
